# Quantified Aortic Luminal Irregularity as a Predictor of Complications and Prognosis After Endovascular Aneurysm Repair

**DOI:** 10.1097/MD.0000000000002863

**Published:** 2016-03-07

**Authors:** Akihiro Hosaka, Masaaki Kato, Manabu Motoki, Hiroko Sugai, Nobukazu Okubo

**Affiliations:** From the Department of Surgery, Tokyo Metropolitan Tama Medical Center, Tokyo (AH), and Department of Cardiovascular Surgery, Morinomiya Hospital, Osaka (MK, MM, HS, NO), Japan.

## Abstract

Atheromatous degeneration of the aorta is considered to be a risk factor for postoperative embolic complications after endovascular treatment, and is associated with a high incidence of vascular events in the long term. We devised a method to quantify the shagginess of the aorta using contrast-enhanced computed tomography (CT) images. This study examined the method's validity and prognostic usefulness in patients undergoing elective endovascular abdominal aortic aneurysm repair (EVAR).

We retrospectively investigated 427 patients who underwent elective EVAR between 2007 and 2013. Preoperative contrast-enhanced CT images with a slice thickness of 1 mm were analyzed using a workstation, and the degree of aortic luminal irregularity from the level of the left subclavian artery ostium to that of the celiac artery ostium was quantified by computing a shagginess score. We compared the computed scores with subjective visual assessments of aortic shagginess. Subsequently, we evaluated the relationship between the computed scores and postoperative prognosis.

The shagginess scores were significantly correlated with the visual assessments of the aortic lumen, which were performed by 5 experienced vascular surgeons (rho ranged from 0.564–0.654, all *P* < 0.001). Multiple logistic regression analysis demonstrated that the shagginess score was independently associated with the development of renal impairment within a month after EVAR (odds ratio, 2.78; 95% confidence interval [CI], 1.83–4.22, *P* < 0.001). The shagginess score was significantly higher in patients who suffered postoperative intestinal and peripheral ischemic complications, as compared with those who did not (*P* < 0.001). The mean postoperative follow-up period was 1207 ± 641 days. Cox proportional hazards regression showed that the shagginess score was a significant independent predictor of all-cause and cardiovascular mortality (hazard ratio [HR], 1.37; 95% CI, 1.09–1.72, *P* = 0.007, and HR, 1.51; 95% CI, 1.04–2.18, *P* = 0.030, respectively).

The results suggest that the shagginess score provides a quantitative reflection of aortic luminal irregularity. It may serve as a useful predictive factor for postoperative renal function deterioration, embolic complications, and long-term mortality after elective EVAR.

## INTRODUCTION

Endovascular aneurysm repair (EVAR) has been established as an effective alternative in the treatment of abdominal aortic and iliac aneurysms. However, EVAR remains challenging in patients with a shaggy aorta, which is an aorta that has extensive atheromatous degeneration with diffuse ulcerations and unstable plaques.^1^ EVAR is difficult in these cases because it entails a high risk of perioperative embolic complications triggered by endoluminal wire and catheter manipulation, which can sometimes lead to fatal conditions.^2–6^

Although it is commonly recognized that patients with a shaggy aorta face high morbidity after endovascular treatment, the embolic potential of aortic atheroma has mainly been evaluated subjectively based on preoperative imaging tests. Few studies have investigated methods of more reliable evaluations of embolic potential by using quantitative and objective assessments of aortic plaque instability. We devised a method to semi-automatically quantify the degree of aortic shagginess on the basis of contrast-enhanced computed tomography (CT) using a 3-dimensional (3D) image analysis workstation. In the present study, we examined the method's validity and evaluated its usefulness as a predictive factor for morbidity and mortality after EVAR.

## METHODS

### Patient Population

The study protocol was approved by the ethics committee of Morinomiya Hospital. Between October 2007 and March 2013, 700 consecutive patients underwent EVAR using a stent graft main body at the Department of Cardiovascular Surgery, Morinomiya Hospital. We excluded patients who had a history of graft replacement or endovascular repair of the aortic arch or descending aorta, those who had a dissected thoracic aorta with a patent false lumen, those who were treated for ruptured or infected aneurysms, and those who did not have preoperative contrast-enhanced CT of the whole aorta performed at our institution and stored in the hospital archive. After making these exclusions, there were 536 remaining patients, each of whom had analyzable preoperative contrast-enhanced CT data and underwent elective EVAR during the study period. We additionally excluded 26 patients who were treated with a fenestrated endovascular graft, 6 patients who were treated with adjunctive bypass grafting to the abdominal aortic branches concurrently with or before EVAR, 61 patients who underwent concomitant renal artery stenting or coverage, and 16 patients with chronic renal failure on maintenance hemodialysis. After these exclusions, 427 patients (343 men and 84 women) were included in the present analysis.

The clinical characteristics of the patients are summarized in Table 1. Patients were assumed to have hypertension or diabetes mellitus if they were taking corresponding medication. Hypercholesterolemia was defined as serum level of total cholesterol ≥220 mg/dl or that of low-density lipoprotein cholesterol ≥140 mg/dl. Coronary artery disease (CAD) was defined as having a history of interventional or surgical treatment of the disease, and cerebrovascular disease (CVD) was diagnosed from a history of stroke. The diagnosis of chronic obstructive pulmonary disease (COPD) was confirmed by respiratory function tests showing that forced expiratory volume in 1 second was less than 70% of forced vital capacity.

**TABLE 1:**
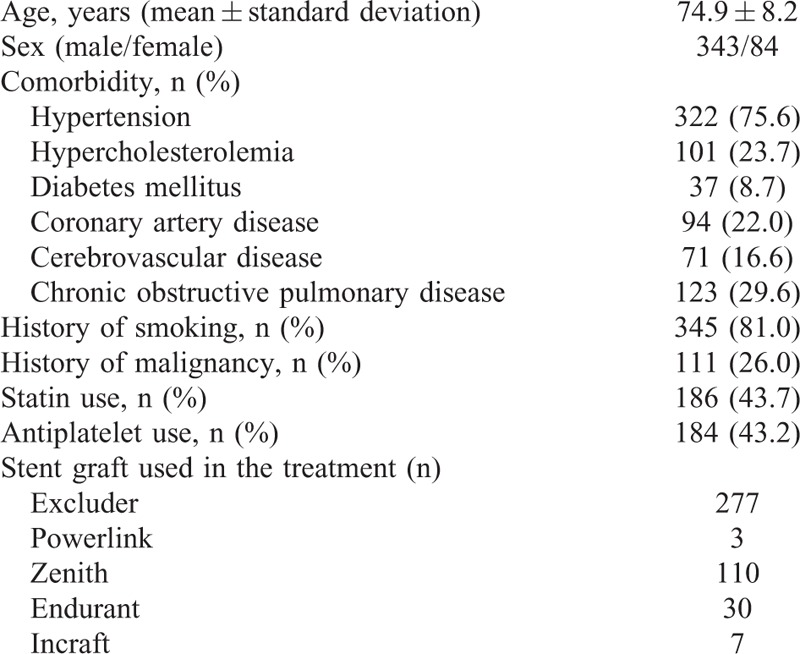
Clinical Characteristics of the Patients

Dual-phase contrast-enhanced CT and/or ultrasonography (in color-flow and pulse-wave modes, without contrast enhancement) were routinely performed within a week postoperatively to evaluate endoleaks and other procedure-related complications.

### Definition of the Shagginess Score

**FIGURE 1:**
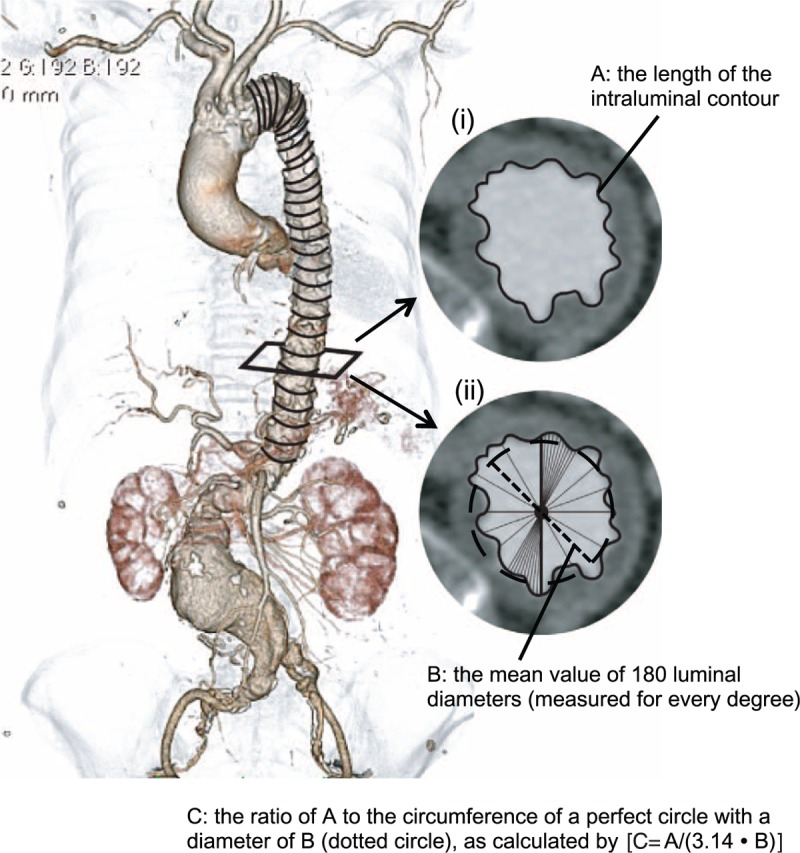
In each multiplanar reconstruction image perpendicular to the automatically defined aortic centerline with a regular slice thickness of 1.2 to 1.7 mm, the contour of the aortic lumen was recognized and traced (i), and its length (A) was calculated. Simultaneously, diameters of the aortic luminal contour through the central luminal line were measured automatically for each individual degree (ii), and the mean value of 180 measurements of the diameters was computed (B, dotted line). Then, the ratio of A to the circumference of a perfect circle with a diameter of B (dotted circle) was determined [C = A/(3.14·B)]. The mean value of (C − 1) × 100 from the left subclavian artery ostium to the celiac artery ostium was calculated and defined as the shagginess score.

All patients included in the present study underwent preoperative contrast-enhanced CT of the whole aorta using a 64-detector row CT scanner (Brilliance 64, Philips Medical Systems, Best, The Netherlands). A bolus-tracking technique was used by placing a circular region of interest within the descending aorta at the level of the tracheal bifurcation, following intravenous administration of 50 to 75 ml contrast agent at 3 to 4 ml/second. The data of CT images of the early arterial phase with a slice thickness of 1 mm were transferred to a 3D image analysis workstation (SYNAPSE VINCENT, Version 4.1, Fujifilm Co., Tokyo, Japan) for evaluation. A central luminal line of the aorta was automatically defined, and multiplanar reconstruction (MPR) images were obtained perpendicularly to the centerline with a regular slice thickness of 1.2 to 1.7 mm from the level of the left subclavian artery to that of the celiac artery. In each MPR image, the contour of the aortic lumen was automatically recognized and traced, and its length (A) was calculated; simultaneously, diameters of the aortic luminal contour through the central luminal line were also measured automatically for every degree, and the mean value of 180 measurements was calculated (B). Then, the ratio of A to the circumference of a perfect circle with a diameter of B was determined [C = A/(3.14·B)] (Figure 1). A is mainly determined by the diameter and irregularity of the aortic lumen. By dividing A by the circumference of a circle with a diameter equal to the average diameter of the lumen (B), we quantified the deviation of the luminal contour from that of a perfect circle. Accordingly, we regarded this ratio (C) as a potential indicator of the degree of luminal irregularity and shagginess.

The shagginess score was then defined as the mean value of (C − 1) × 100 from the level just distal to the left subclavian artery ostium to that just above the celiac artery ostium. In practice, the process of calculating the score required a few minutes.

### Shagginess Score and Grading of Aortic Shagginess by Visual Assessment

Among the study population, 54 patients underwent EVAR during the period between January and December 2011, and their preoperative axial CT images were reviewed independently by 5 experienced vascular surgeons. The observers visually graded the irregularity of the aortic luminal surface of each patient on a scale of 1 to 10 (1 = clean and not atheromatous, 10 = most severely shaggy) after being given reference sample images. The correlations between the visual scales and shagginess scores were evaluated.

### Postoperative Renal Dysfunction, Ischemic Events, and All-Cause and Cardiovascular Mortality

The relationships between the pre- and intraoperative risk factors (including the shagginess score) and postoperative renal dysfunction, ischemic complications, and long-term mortality were evaluated. Data on the serum creatinine levels of each patient were collected before surgery, throughout the perioperative period, and at a 1-month follow-up. Postoperative renal impairment was defined as an absolute increase in serum creatinine of ≥0.3 mg/dl or a percentage increase in serum creatinine of ≥50% within a month after surgery, as compared with the preoperative level.^7^ The diagnosis of bowel ischemia was established using colonic endoscopy and/or contrast-enhanced CT performed following characteristic clinical presentations, such as abdominal pain and melena. Blue toe syndrome was diagnosed from the clinical findings. Deaths and causes of mortality were identified by chart review and by direct contact with patients and their families, if necessary.

### Statistical Analysis

Continuous data are presented as means ± standard deviations (SDs). The Mann–Whitney *U* test was used for comparisons between groups of patients, and relationships between categorical variables were compared using Chi-squared test. Correlation analyses were performed using the Spearman rank test. Multiple logistic regression analysis was performed to determine the relative contributions of variables associated with postoperative renal impairment. Cox proportional hazards analyses were used to examine factors related to all-cause and cardiovascular mortality. Each multivariate analysis included all variables that were significant in univariate analyses at the *P* values <0.1 level. All analyses were performed using PASW Statistics 18 (SPSS, Inc., Chicago, IL). *P* values less than 0.05 were regarded as indicating statistical significance.

## RESULTS

### Profiles of the Shagginess Score

The primary technical success rate was 94.8%; minor type I or III endoleaks were observed in 22 patients at completion angiography. In 20 of these patients, the endoleaks had resolved by the time of the follow-up CT and/or duplex echography that were performed 4 to 6 days after EVAR. Postoperative 30-day mortality was 0.2%. The mean shagginess score was 8.254 ± 0.665, with a range of 7.013 to 12.441. The shagginess score was significantly higher in patients with hypertension, CAD, and COPD (*P* < 0.001, 0.036, and 0.028, respectively), but did not differ significantly according to sex, or between patients with and without a history of hypercholesterolemia, diabetes, CVD, or smoking (Table 2). The shagginess score did not show correlations with age or preoperative serum creatinine level (rho = 0.236 and 0.194, respectively, both *P* < 0.001).

**TABLE 2:**
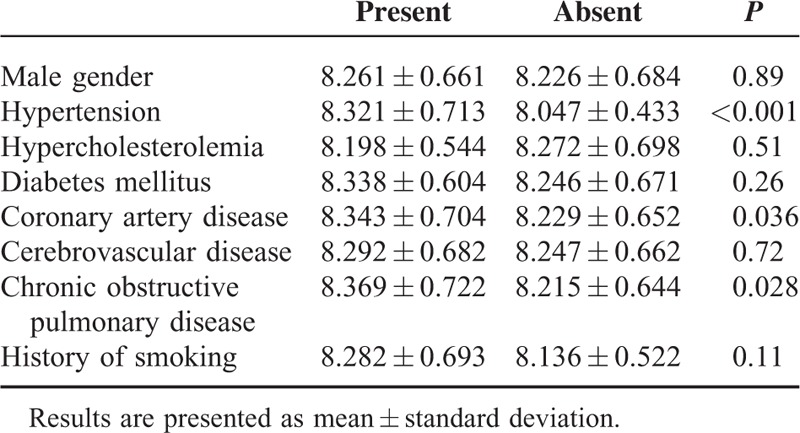
Shagginess Score and Preoperative Risk Factors for Atherosclerosis

**FIGURE 2:**
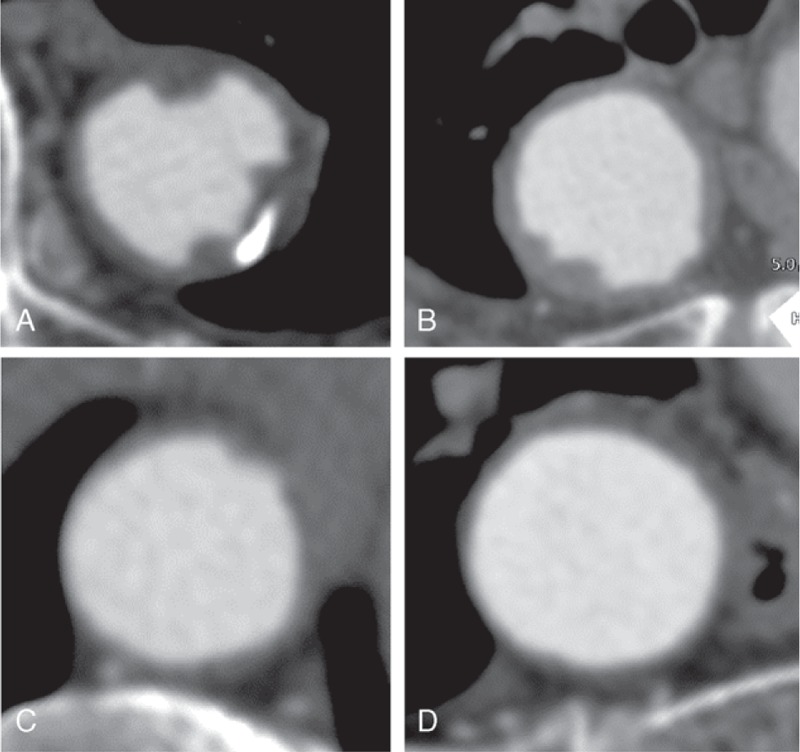
Examples of multiplanar reconstruction computed tomography images perpendicular to the aortic centerline. (A) A patient with both hypertension and coronary artery disease (CAD) (shagginess score, 12.434). (B) A patient with CAD and without hypertension (shagginess score, 8.538). (C) A patient with hypertension and without CAD (shagginess score, 8.133). (D) A patient without hypertension or CAD (shagginess score, 7.705).

The CT images and shagginess scores of several patients are shown in Figure 2. The shagginess score showed a significant correlation with the visual assessments of the aortic lumen, which were made on a 10-point scale by 5 observers (rho = 0.564, 0.595, 0.601, 0.627, and 0.654, respectively, all *P* < 0.001).

### Shagginess Score, Postoperative Renal Impairment, and Ischemic Events

We evaluated the relationships between the pre- and intraoperative conditions and change in renal function within the first postoperative month. Seven patients were excluded from the analysis because of a lack of data. Postoperative renal impairment was observed in 70 patients. Univariate analyses demonstrated that the shagginess score, the preoperative level of serum creatinine, and the amount of contrast medium used during the operation were significantly higher in patients who suffered postoperative renal impairment, as compared with those who did not. Patients with postoperative renal impairment were significantly older and more likely to have hypertension. A multiple logistic regression analysis showed that the shagginess score, the preoperative serum creatinine level, and the amount of contrast medium used intraoperatively were independently associated with the development of renal impairment after EVAR (Table 3).

**TABLE 3:**
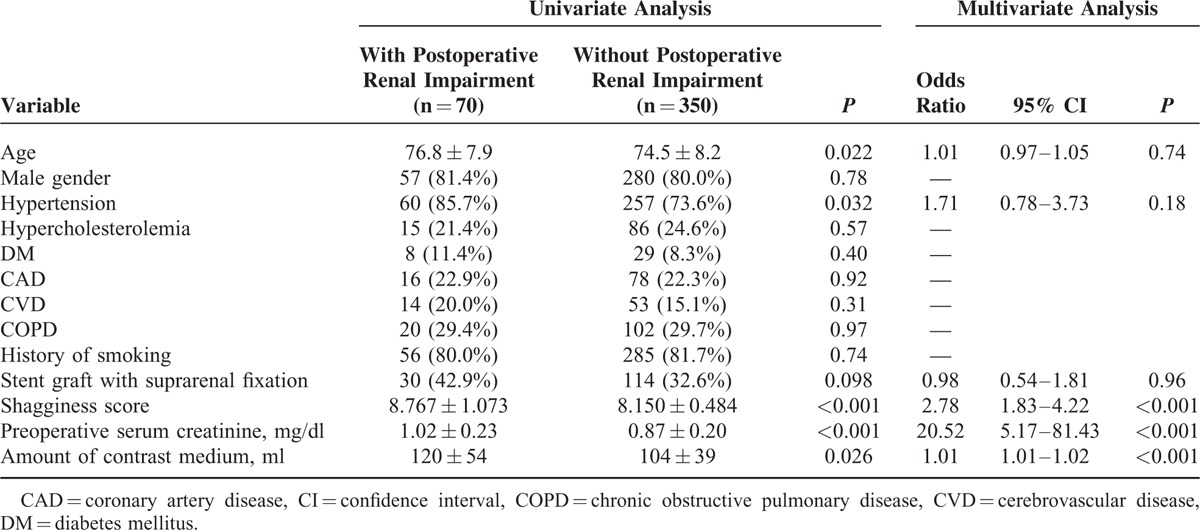
Pre- and Intraoperative Conditions and Postoperative Renal Impairment

Two patients developed postoperative bowel ischemia, 3 developed blue toe syndrome, and 1 developed both; 4 of these 6 patients’ cases were also complicated by postoperative renal impairment. The shagginess score was significantly higher in patients with bowel ischemia and/or blue toe syndrome after EVAR, as compared with those who did not develop these complications (*P* < 0.001).

### Shagginess Score, All-Cause Mortality, and Cardiovascular Mortality

The mean follow-up period was 1207 ± 641 days, with a range of 3 to 2570 days. Ninety-one patients died during the study period, of whom 26 died of a cardiovascular cause. A Cox proportional hazards model demonstrated that the shagginess score was a significant independent predictor of all-cause mortality (hazard ratio [HR], 1.37; 95% confidence interval [CI], 1.09–1.72, *P* = 0.007), as were age at operation (HR, 1.09; 95% CI, 1.05–1.12, *P* < 0.001) and history of malignancy (HR, 2.48; 95% CI, 1.59–3.86, *P* < 0.001) (Table 4). With respect to cardiovascular mortality, the independent predictive factors with statistical significance were shagginess score (HR, 1.51; 95% CI, 1.04–2.18, *P* = 0.030), age at operation (HR, 1.09; 95% CI, 1.03–1.15, *P* = 0.004), and preoperative level of serum creatinine (HR, 6.35; 95% CI, 1.17–34.54, *P* = 0.032) (Table 5).

**TABLE 4:**
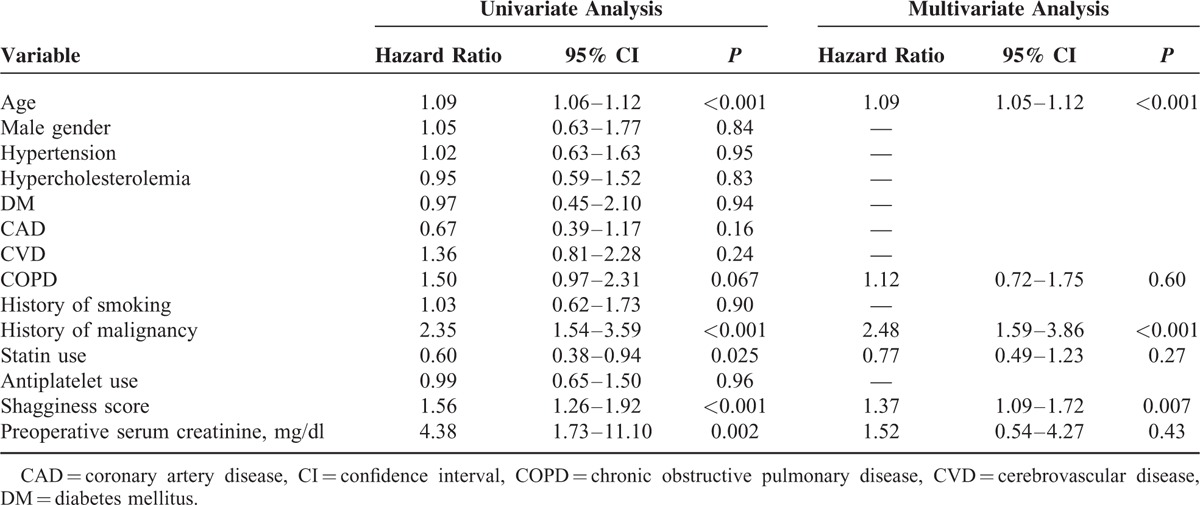
Preoperative Conditions and All-Cause Mortality

**TABLE 5:**
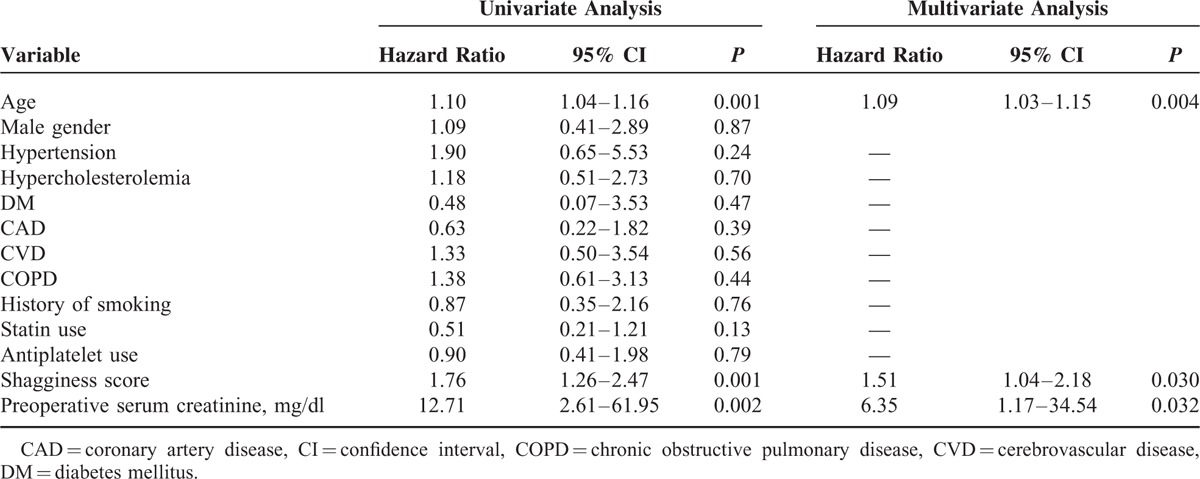
Preoperative Conditions and Cardiovascular Mortality

## DISCUSSION

The shagginess score is an assessment of aortic shagginess that can be calculated semi-automatically from the contrast-enhanced CT images within a processing time of a few minutes. The results of the present study demonstrated that the shagginess score corresponded well with the visual assessment of aortic luminal irregularity. The score showed significant association with the occurrence of postoperative renal, mesenteric, and peripheral arterial complications mostly attributable to atheroembolism. Further, our results suggest that the score may have a prognostic value in predicting long-term mortality, indicating its potential usefulness in determining treatment strategies for EVAR candidates. To the best of our knowledge, this is the first attempt to quantitatively characterize the shagginess of the aorta and to evaluate the clinical value of the proposed index.

Vulnerable plaques are widely recognized to play important roles in the development of cardiovascular events. Efforts have been made to identify these lesions in the coronary and carotid arteries, and to quantify their instability using various imaging modalities, such as ultrasonography, CT, magnetic resonance imaging (MRI), positron emission tomography (PET), and optical coherence tomography.^8–14^ The presence of unstable plaques in the aorta is also considered a risk factor for distal atheroembolism, and previous studies have demonstrated the usefulness of various grading scales of aortic atheroma, as determined using transesophageal echocardiography (TEE), contrast-enhanced CT, and PET.^5,6,15–20^ Among these modalities, TEE is most commonly used in the evaluation of aortic plaques. However, TEE has several limitations—it requires operators with a certain level of skills and sufficient time to examine the whole thoracic aorta. PET can quantify the inflammatory activity of the plaques, but it is less available for routine use as a screening tool. On the other hand, CT provides unbiased information on the whole aorta in a single rapid scan, and its advantages have recently been broadened by the development of the multidetector apparatus and image analysis software.

Previously suggested methods of assessing aortic shagginess have used either TEE or contrast-enhanced CT, and have mainly focused on thickness and degree of ulceration, protrusion, and mobility of the atheroma.^5,6,15–19^ Katsanos et al^18^ defined complex atheromatous plaques as present if the lesions evaluated by TEE were >4 mm thick, ulcerated, or had mobile components. In a meta-analysis, they reported that the presence of complex plaques in the descending aorta can be a risk factor for stroke. Ko et al^19^ defined high-risk aortic atherosclerotic disease as the presence of aortic plaque in the ascending aorta and aortic arch that had a thickness of ≥4 mm, was ulcerated, or was hypoattenuated on contrast-enhanced CT. They demonstrated that high-risk disease was prevalent in patients with embolic stroke. Several studies have investigated the impact of aortic shagginess on the occurrence of embolic complications associated with EVAR. Patel et al^5^ graded the degree of aortic luminal irregularity on a scale of 0 to 3, while Toya et al^6^ defined a shaggy aorta as the presence of a fragile atheroma >4 mm thick; both of these definitions were based on CT images reviewed by experts. Both studies showed that aortic shagginess was a significant predictor of postoperative ischemic events. Although these classifications of aortic plaques have proven significance, they mainly depend on the experts’ visual assessments of images acquired by TEE or CT. This entails the potential for variation that result from the inherently subjective nature of the evaluations. In addition, any detailed visual evaluation of the acquired images requires considerable time.

We considered that the ulcerated or protruding fragile aortic atheroma results in the elongation of the luminal contour of the aorta, as assessed in a cross-sectional CT image orthogonal to the central luminal line. Further, we considered that the difference between its length and a corresponding perfect circle might reflect the morphological irregularity of the aortic surface. The shagginess score can be calculated semi-automatically within a few minutes by manually determining the proximal and distal ends of the target artery. Our automated method using a standard contrast-enhanced CT could objectively quantify the degree of aortic shagginess, considering the close correlation with the visual assessment.

Postoperative renal impairment following EVAR is multifactorial, and previous studies have indicated potential contributing factors that include preexisting renal insufficiency, fixation level of the stent graft, nephrotoxicity of contrast medium, and procedure-related microembolism.^21–26^ In concordance with previous reports,^21^ the results of the present study demonstrated that preexistent renal dysfunction was a significant risk factor. The impact of fixation type on renal function remains controversial^22–26^; we found that the use of a device with suprarenal fixation was not independently associated with the deteriorated renal function after EVAR in the short term. Nephrotoxicity of contrast medium is considered to play a certain role, but there is little information on the precise relationship between the volume of contrast medium that is used during EVAR and postoperative kidney function. Our study showed that this volume was an independent risk factor, as previously indicated in coronary interventions.^27^ Microembolism caused by dislodged thrombus from the proximal aortic surface during the procedure has been suggested as an important etiologic factor.^22,24,25^ The shagginess score was significantly associated with the postoperative renal impairment and other ischemic complications in the present study, which might indicate that it reflects the instability of aortic plaque and predisposition to embolic events. The score can potentially be a useful diagnostic index to evaluate the risk of postoperative atheroembolic complications in patients undergoing EVAR.

Previous studies have suggested that the severity of atheromatous degeneration of the thoracic aorta could be predictive of future vascular events.^15–17^ Sen et al^16^ demonstrated that patients with atheroma progression in the aortic arch had a high risk of recurrent vascular events. Izumi et al^17^ reported that those with severe atheromatous plaques in the thoracic aorta had a lower survival rate and higher embolic event rate, as compared with those without these plaques. Complex plaques in the aorta can be considered a marker of systemic atherosclerotic disease, reflecting high cardiovascular risk.^18^ The present study demonstrated that the shagginess score was an independent risk factor for all-cause and cardiovascular mortality after EVAR, over a mean follow-up period of 3.3 years. These findings suggest that the score might be representative of systemic atherosclerotic burden and be indicative of long-term prognosis. It would be interesting to elucidate its clinical significance in a broader population with a various degree of risks for atherosclerosis in the longer term.

The present study has some limitations. Because of its retrospective design, all patients were not systematically followed up (although most of them were on a uniform postoperative follow-up protocol). Further, unmeasured confounding factors might have affected the results of the study. In addition, patient selection bias might exist, since this was a single-center study. Additional studies are necessary to delineate the clinical usefulness of the shagginess score.

## CONCLUSIONS

The shagginess score appears to quantitatively characterize aortic luminal irregularity. It could serve as a useful diagnostic parameter for predicting treatment-associated atheroembolic complications and long-term prognosis in patients undergoing EVAR. Larger and longer-term studies are necessary in order to further clarify the clinical value of the score.

## References

[1] Hollier LH Kazmier FJ Ochsner J “Shaggy” aorta syndrome with atheromatous embolization to visceral vessels. *Ann Vasc Surg* 1991; 5:439–444. 1958458 10.1007/BF02133048

[2] Dadian N Ohki T Veith FJ Overt colon ischemia after endovascular aneurysm repair: the importance of microembolization as an etiology. *J Vasc Surg* 2001; 34:986–996. 11743550 10.1067/mva.2001.119241

[3] Maldonado TS Rockman CB Riles E Ischemic complications after endovascular abdominal aortic aneurysm repair. *J Vasc Surg* 2004; 40:703–709.discussion 709–710. 15472598 10.1016/j.jvs.2004.07.032

[4] Miller A Marotta M Scordi-Bello I Ischemic colitis after endovascular aortoiliac aneurysm repair: a 10-year retrospective study. *Arch Surg* 2009; 144:900–903. 19841356 10.1001/archsurg.2009.70

[5] Patel SD Constantinou J Hamilton H Editor's choice—a shaggy aorta is associated with mesenteric embolisation in patients undergoing fenestrated endografts to treat paravisceral aortic aneurysms. *Eur J Vasc Endovasc Surg* 2014; 47:374–379. 24502998 10.1016/j.ejvs.2013.12.027

[6] Toya N Baba T Kanaoka Y Embolic complications after endovascular repair of abdominal aortic aneurysms. *Surg Today* 2014; 44:1893–1899. 24276152 10.1007/s00595-013-0795-y

[7] Mehta RL Kellum JA Shah SV Acute Kidney Injury Network: report of an initiative to improve outcomes in acute kidney injury. *Crit Care* 2007; 11:R31. 17331245 10.1186/cc5713 PMC2206446

[8] Rosa GM Bauckneht M Masoero G The vulnerable coronary plaque: update on imaging technologies. *Thromb Haemost* 2013; 110:706–722. 23803753 10.1160/TH13-02-0121

[9] Saremi F Achenbach S Coronary plaque characterization using CT. *AJR Am J Roentgenol* 2015; 204:W249–W260. 25714309 10.2214/AJR.14.13760

[10] Varma N Hinojar R D’Cruz D Coronary vessel wall contrast enhancement imaging as a potential direct marker of coronary involvement: integration of findings from CAD and SLE patients. *JACC Cardiovasc Imaging* 2014; 7:762–770. 25051945 10.1016/j.jcmg.2014.03.012 PMC4136741

[11] Kato K Yonetsu T Kim SJ Nonculprit plaques in patients with acute coronary syndromes have more vulnerable features compared with those with non-acute coronary syndromes: a 3-vessel optical coherence tomography study. *Circ Cardiovasc Imaging* 2012; 5:433–440. 22679059 10.1161/CIRCIMAGING.112.973701

[12] Hoogi A Adam D Hoffman A Carotid plaque vulnerability: quantification of neovascularization on contrast-enhanced ultrasound with histopathologic correlation. *AJR Am J Roentgenol* 2011; 196:431–436. 21257897 10.2214/AJR.10.4522

[13] Zavodni AE Wasserman BA McClelland RL Carotid artery plaque morphology and composition in relation to incident cardiovascular events: the Multi-Ethnic Study of Atherosclerosis (MESA). *Radiology* 2014; 271:381–389. 24592924 10.1148/radiol.14131020 PMC4263652

[14] Saito H Kuroda S Hirata K Validity of dual MRI and F-FDG PET imaging in predicting vulnerable and inflamed carotid plaque. *Cerebrovasc Dis* 2013; 35:370–377. 23635390 10.1159/000348846

[15] The French Study of Aortic Plaques in Stroke Group. Atherosclerotic disease of the aortic arch as a risk factor for recurrent ischemic stroke. *N Engl J Med* 1996; 334:1216–1221. 8606716 10.1056/NEJM199605093341902

[16] Sen S Hinderliter A Sen PK Aortic arch atheroma progression and recurrent vascular events in patients with stroke or transient ischemic attack. *Circulation* 2007; 116:928–935. 17684150 10.1161/CIRCULATIONAHA.106.671727

[17] Izumi C Takahashi S Miyake M Impact of aortic plaque morphology on survival rate and incidence of a subsequent embolic event—long-term follow-up data—. *Circ J* 2010; 74:2152–2157. 20689218 10.1253/circj.cj-10-0414

[18] Katsanos AH Giannopoulos S Kosmidou M Complex atheromatous plaques in the descending aorta and the risk of stroke: a systematic review and meta-analysis. *Stroke* 2014; 45:1764–1770. 24788969 10.1161/STROKEAHA.114.005190

[19] Ko Y Park JH Yang MH Significance of aortic atherosclerotic disease in possibly embolic stroke: 64-multidetector row computed tomography study. *J Neurol* 2010; 257:699–705. 19937048 10.1007/s00415-009-5391-0

[20] Rudd JH Myers KS Bansilal S (18)Fluorodeoxyglucose positron emission tomography imaging of atherosclerotic plaque inflammation is highly reproducible: implications for atherosclerosis therapy trials. *J Am Coll Cardiol* 2007; 50:892–896. 17719477 10.1016/j.jacc.2007.05.024

[21] Kim M Brady JE Li G Anesthetic technique and acute kidney injury in endovascular abdominal aortic aneurysm repair. *J Cardiothorac Vasc Anesth* 2014; 28:572–578. 24321848 10.1053/j.jvca.2013.06.001

[22] Krämer SC Seifarth H Pamler R Renal infarction following endovascular aortic aneurysm repair: incidence and clinical consequences. *J Endovasc Ther* 2002; 9:98–102. 11958332 10.1177/152660280200900116

[23] Walsh SR Boyle JR Lynch AG Suprarenal endograft fixation and medium-term renal function: systematic review and meta-analysis. *J Vasc Surg* 2008; 47:1364–1370. 18280095 10.1016/j.jvs.2007.11.029

[24] Saratzis A Sarafidis P Melas N Suprarenal graft fixation in endovascular abdominal aortic aneurysm repair is associated with a decrease in renal function. *J Vasc Surg* 2012; 56:594–600. 22579136 10.1016/j.jvs.2012.01.078

[25] Antonello M Menegolo M Piazza M Outcomes of endovascular aneurysm repair on renal function compared with open repair. *J Vasc Surg* 2013; 58:886–893. 23688627 10.1016/j.jvs.2013.02.249

[26] Kouvelos GN Boletis I Papa N Analysis of effects of fixation type on renal function after endovascular aneurysm repair. *J Endovasc Ther* 2013; 20:334–344. 23731306 10.1583/12-4177MR.1

[27] Laskey WK Jenkins C Selzer F Volume-to-creatinine clearance ratio: a pharmacokinetically based risk factor for prediction of early creatinine increase after percutaneous coronary intervention. *J Am Coll Cardiol* 2007; 50:584–590. 17692741 10.1016/j.jacc.2007.03.058

